# Letter to the Editor: Mediating effect of lower extremity muscle on the relationship between obesity and osteoarthritis in middle-aged and elderly women in Korea: based on the 2009–2011 Korea National Health and Nutrition Examination Survey

**DOI:** 10.4178/epih.e2024091

**Published:** 2024-11-22

**Authors:** Jinyue Chen, Haozhu Chen

**Affiliations:** 1 Renhuai People’s Hospital, Renhuai, China; 2 Hubei University of Chinese Medicine, Wuhan, China

Dear Editor,

The article “Mediating effect of lower extremity muscle on the relationship between obesity and osteoarthritis in middle-aged and elderly women in Korea: based on the 2009–2011 Korea National Health and Nutrition Examination Survey,” authored by Kim et al. [1], published in “*Epidemiology and Health*,” explores the mediating role of the lower extremity muscle index (LMI) in the relationship between obesity and knee osteoarthritis (KOA) among middle-aged and elderly women in Korea. This investigation utilized data from the 2009–2011 Korean National Health and Nutrition Examination Survey. The study’s findings reveal that the prevalence of KOA is significantly higher in the obese and central obesity groups compared to the normal group, and it varies with different levels of LMI. Mediation analysis showed that LMI partially mediates the relationship between body mass index (BMI) and Kellgren-Lawrence (KL) grading, as well as between waist circumference (WC) and KL grading, accounting for 4.8% and 6.7% of the total effects of BMI and WC on KL grading, respectively. This study provides a new perspective on the relationship between obesity and KOA, highlighting the potential protective role of lower extremity muscle mass. The findings suggest that enhancing leg muscle strength could partially mitigate the risk of KOA associated with obesity.

While recognizing the value of this study, we would like to offer the following comments. Given its cross-sectional design, this study can only suggest an association between LMI and the relationship between obesity and KOA among middle-aged and elderly women; it cannot establish causality. Therefore, we recommend that future research employ Mendelian randomization to further investigate the causal links between LMI and the aforementioned conditions [2].

The authors included a variety of covariates such as age, average monthly household income, education level, marital status, employment status, region, smoking, alcohol consumption, lack of exercise, energy and protein intake, self-reported health status, menopause, and comorbidities to adjust for potential confounding factors, which is commendable. However, despite these multivariable adjustments, there may still be unadjusted confounding factors that could influence the relationship between LMI, obesity, and KOA. Therefore, we suggest further expanding the range of covariates. Considering additional factors such as diet [3], physical activity [4], sleep patterns [5], and bone density [6], may enhance the stability and reliability of the results by providing a more comprehensive assessment of the factors influencing the association between LMI and KOA. This could further strengthen the credibility of the study’s conclusions.

In summary, this study represents high-quality research that delivers a comprehensive and in-depth analysis of the association between LMI and the relationship between obesity and KOA in middle-aged and elderly women. This study undoubtedly provides new insights into the complex interplay between obesity and KOA, potentially influencing the development of innovative prevention and treatment strategies. Such advancements could have a significant impact on clinical practice, public health policy, and future research directions. Our suggestions aim to further strengthen an already exceptional research outcome, and we look forward to the authors’ continued contributions to high-quality research in the future.

## Ethics statement

This article does not contain any studies with human participants or animals performed by any of the authors.
